# *MIF -173*G/C (rs755622) polymorphism modulates coronary artery disease risk: evidence from a systematic meta-analysis

**DOI:** 10.1186/s12872-020-01564-4

**Published:** 2020-06-19

**Authors:** De-Yang Li, Jin-Yu Zhang, Qing-Jie Chen, Fen Liu, Qian Zhao, Xiao-Ming Gao, Xiao-Mei Li, Yi-Ning Yang

**Affiliations:** 1 State Key Laboratory of Pathogenesis, Prevention and Treatment of High Incidence Diseases in Central Asia, Department of Cardiology, First Affiliated Hospital of Xinjiang Medical University, Urumqi, China; 2 Department one of coronary heart disease, First Affiliated Hospital of Xinjiang Medical University, Urumqi, 830011 China; 3 Rehabilitation department, First Affiliated Hospital of Xinjiang Medical University, Urumqi, China

**Keywords:** Meta-analysis, Macrophage migration inhibitory factor, Polymorphism, Coronary artery disease

## Abstract

**Background:**

Coronary artery disease (CAD) remains one of the major causes of death in humans. Genetic testing may allow early detection and prevention of this disease. This study aimed to investigate the association between the macrophage migration inhibitory factor (*MIF*) -173G/C (rs755622) polymorphism and susceptibility to CAD based on a meta-analysis.

**Methods:**

We searched several databases to identify observational case-control studies investigating the association between the *MIF* -173G > C (rs755622) polymorphism and CAD risk published before July 30, 2019. Data were analyzed using the STATA software.

**Results:**

Six studies, comprising a total of 1172 CAD cases and 1564 controls evaluated for *MIF* polymorphisms, were included. The occurrence of CAD was found to be associated with the C allele of the *MIF* rs755622 SNP in the total population (C/G, OR = 1.489, 95% CI = 1.223–1.813). Further, *MIF* –173G/C polymorphism was significantly associated with CAD under the allelic model in the Asian (C/G, OR = 1.775, 95% CI = 1.365–2.309) and Caucasian (C/G, OR = 1.288, 95% CI 1.003–1.654) subgroups. The data showed that the risk of CAD was higher in the population carrying the C allele.

**Conclusions:**

We found evidence of associations between *MIF* -173C/G and CAD susceptibility in the Asian and Caucasian populations.

## Background

Atherosclerosis is a chronic arterial wall inflammatory disease [1] associated with endothelial dysfunction, intimal hyperplasia, smooth muscle hyperplasia, lipid deposition, plaque formation, and micro-vein formation. Endothelial dysfunction is associated with the expression of chemokines and adhesion molecules, such as vascular cell adhesion molecule-1 [2]. Cytokines produced in the plaque micro-environment, such as interleukin (IL)-1, IL-6, IL-8, and tumor necrosis factor (TNF), trigger the recruitment of inflammatory cells [3]. Chemokines are released by endothelial cells, mastocytes, platelets, macrophages, and lymphocytes [4]. They mediate the migration of leukocytes to inflamed tissues and control the inflammatory reactions in various immune-mediated diseases. Chemokine expression is associated with atherosclerotic lesion development and vascular remodeling [5]. Owing to such inflammatory reactions and the accumulation of blood lipids, the artery becomes less elastic and narrower, promoting an atherosclerotic plaque formation. When vascular plaques occur in the coronary artery of the heart, they lead to a coronary artery disease (CAD) [1]. In recent years, the macrophage migration inhibitory factor (*MIF*) has been extensively studied in molecular functional and genetics studies. In 1966, *MIF* was identified as a soluble factor secreted by T cells that delayed hypersensitivity reactions and inhibited the random migration of macrophages [4]. Later studies revealed that MIF is stored in the pituitary gland and secreted during endotoxemia and plays a key regulatory role in innate immunity by counter-regulating glucocorticoids [6]. It has been currently considered to act as a chemokine-like multidirectional inflammatory cytokine and has been recognized to mediate numerous acute and chronic inflammatory diseases [7]. *MIF* is quite ubiquitously expressed and is an upstream immunomodulatory cytokine. It plays an important role in promoting inflammatory responses in CAD [8], diabetes [9], rheumatoid arthritis [10], septicemia [11], psoriasis [12], and other diseases. The *MIF* gene is located on chromosome 22q11.2, and two functional promoter polymorphisms have been studied [13]. One is a G-to-C transition at − 173 (rs755622) and the other is a (CATT)_5–8_ tetranucleotide repeat at − 794. The *MIF* − 173C allele creates a putative binding site for the transcription factor activating enhancer binding protein 4 and is associated with increased *MIF* gene expression and protein levels in a cell-type-dependent manner [14].

However, according to current reports, *MIF* − 173C/G is closely related to the production and expression of *MIF*, which causes the occurrence and development of CAD by promote the inflammatory response.

Some studies comparing *MIF* − 173C/G have found associations with CAD pathogenesis. To overcome the limitations and outcome bias of individual studies, and to address the inconsistencies in the findings among the various studies, we conducted a more comprehensive meta-analysis based on a systematic literature review to confirm whether *MIF* − 173C/G was associated with increased sensitivity and risk for CAD.

## Methods

### Data search and selection

We searched for reports on the *MIF* − 173G/C (rs755622) polymorphism in the PubMed, Embase, WanFang, China National Knowledge Infrastructure databases using the search terms “macrophage migration inhibitory factors,” or “*MIF*” or “rs755622” or “-173G/C,” and “genetic polymorphism” or “gene polymorphism” and “coronary artery disease,” or “myocardial infarction” in various combinations to identify studies that had investigated the association between *MIF* -173G/C and CAD, with the last search being conducted on June 14, 2019. No language restrictions were applied. In addition, we reviewed all references cited in the obtained papers, to identify additional studies that were not included in the above-mentioned electronic databases.

### Inclusion/exclusion criteria

Studies were included if they met the following criteria: (1) assessment of the association between *MIF* -173 G > C and CAD risk, (2) case-control studies, and (3) sufficient genotype frequencies for calculating the odds ratio (OR) and 95% confidence interval (CI). (4) CAD was diagnosed according to the 1979 WHO criteria for CAD diagnosis: at least one major vessel with ≥50% stenosis in coronary angiography. Studies were excluded if they (1) were a duplication of a previous publication; (2) were comments, abstracts, overlapping studies, reviews, or editorial comments; (3) were family-based studies of pedigrees; (4) had no detailed genotype data and other information; and (5) had a cohort size of less than 100 subjects.

### Data extraction

We extracted the following information from the selected articles: author, year of publication, country/countries, ethnicity, number of cases and controls, and genotyping methods used. In addition, we determined whether data had been analyzed correctly.

### Quality assessment

We used the Newcastle-Ottawa scale to evaluate the quality of the studies included [15] (www.liebertpub.com/gtmb, Supplementary Fig. S1). Each study is scored for 8 items, and the scores are added up to obtain the final score, which ranges from 0 to 10 points, with a score of ≥7 points indicating a high-quality study. All authors reviewed and scored the studies. Five studies included [16–20] had a score of ≥7 points. One study by Bin et al. [21] on the relationship between gene polymorphism and CAD in smokers scored only 6 points, but we included this study as this score was expected to have a limited influence on the final analysis results.

### Data analyses

We used Chi-squared tests to evaluate population genotype frequencies to test for the Hardy-Weinberg equilibrium (HWE), if *P* ≥ 0.05, indicate population gene genetic balance. The OR and 95% CI were calculated by the same method to evaluate the risk of CAD in different genes, with *P* < 0.05 indicating a significant effect. We used the Cochran’s Q test statistic and the *I*^*2*^ statistic to assess variation and within- and between- study heterogeneities [22]. Q-test results may be unreliable when a small number of studies is included in the meta-analysis. Therefore, a heterogeneity with *P* < 0.1 indicates the presence of heterogeneity [23]. We used *I*^*2*^ (*I*^*2*^ = 100% × (Q – df)/Q) to assess the heterogeneity of the studies included [11]. *I*^*2*^ is an index to evaluate the degree of inconsistency among the studies included and to test whether the percentage total variation among the studies was due to heterogeneity or by chance. *I*^*2*^ values range from 0 to 100%, and *I*^*2*^ values of 25, 50, and 75% were considered to indicate low, moderate, and high heterogeneity, respectively. When *I*^*2*^ is greater than 50%, heterogeneity cannot be ignored, and subgroup analysis is needed to evaluate the source of heterogeneity [24]. We assessed the association between *MIF* -173G/C and CAD under six genetic models: an allelic model (C vs. G), homozygote model (CC vs. GG), a heterozygote model (CG vs. CC), a recessive model (CC vs. CG + GG), a dominant model (GG vs. CG + CC), and an additive model (CG vs. CC + GG). To find ethnic effects, we divided patients into two subgroups, Asians and Caucasians, and we conducted a sensitivity analysis by successively omitting one of the six studies and assessing the effect thereof on the results. Begg’s funnel plots and Egger’s linear regression test were used to assess for publication bias across studies [25]. Statistical analyses were performed using the STATA 12.0 software (StataCorp, College Station, TX).

## Results

### Baseline characteristics of eligible studies

**Fig. 1:**
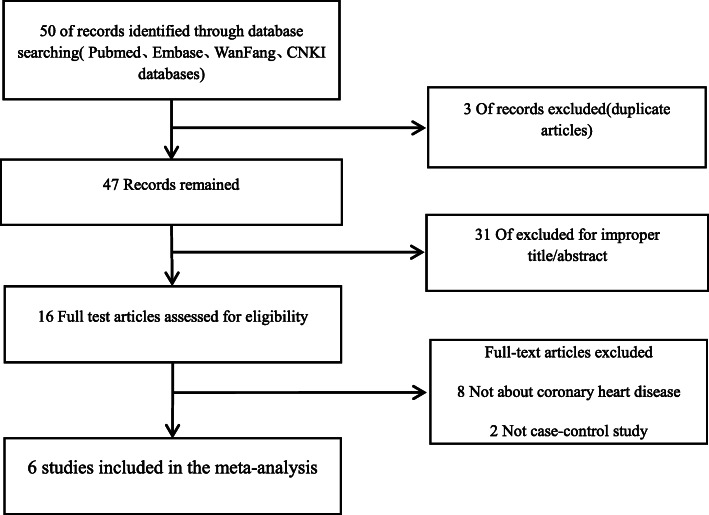
Flowchart of study selection

We found 50 studies based on electronic and manual searches. Three studies were excluded because they reported duplicate data, 31 were excluded from full-text review based on the title and abstract. Sixteen full-text articles were assessed for eligibility. Among these, eight were not about CAD, two were not case-control studies, and thus, these studies were excluded. The six reports that met our inclusion criteria were included into our meta-analysis [16–21]. Details on the selection and exclusion processes have been shown in the flow chart in Fig. 1. Together, the six studies comprised 1172 patients with CAD and 1564 controls evaluated for *MIF* polymorphisms (Table 1). All studies examined the *MIF* -173C/G polymorphism. Characteristic features of the studies were assessed in the meta-analysis.

**Table 1:**
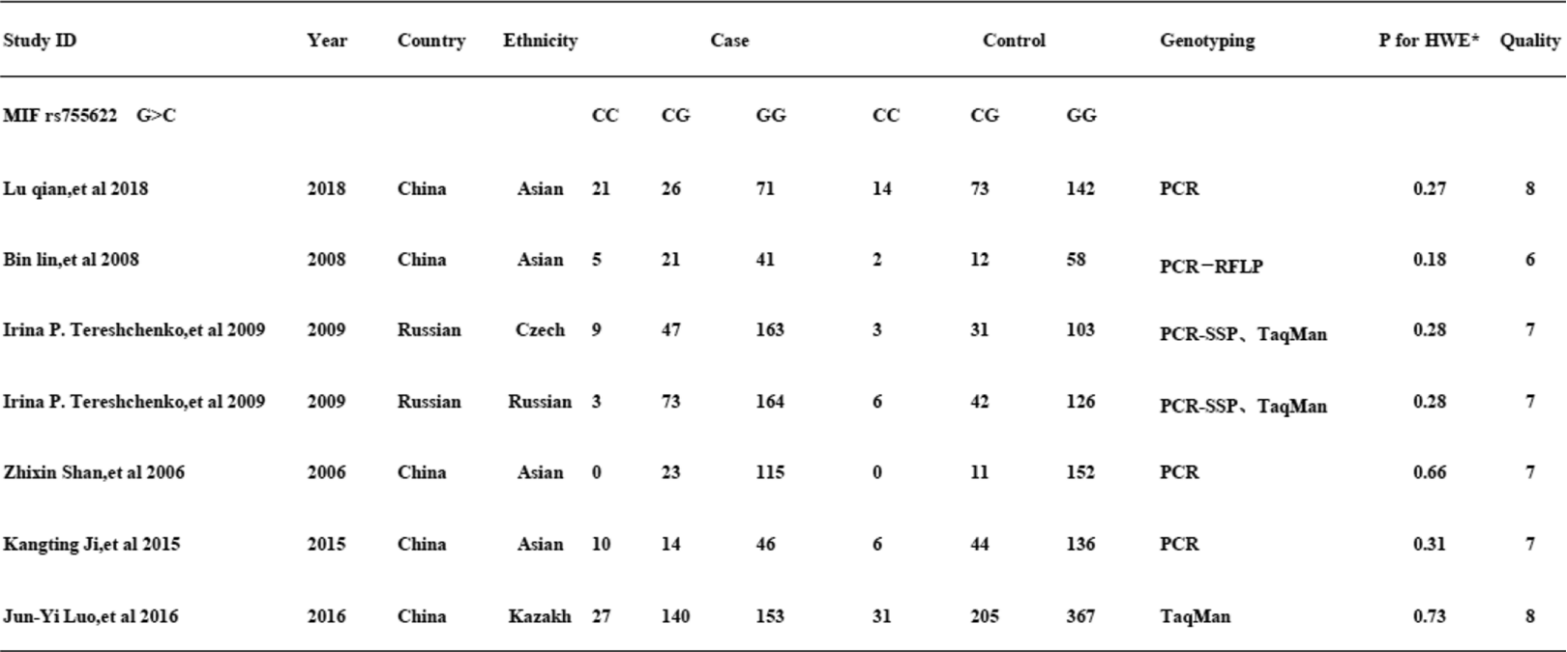
Characteristic of studies inckuded in the meta-analysis

### Meta-analysis of the association between *MIF* -173 C/G and CAD susceptibility

**Fig. 2:**
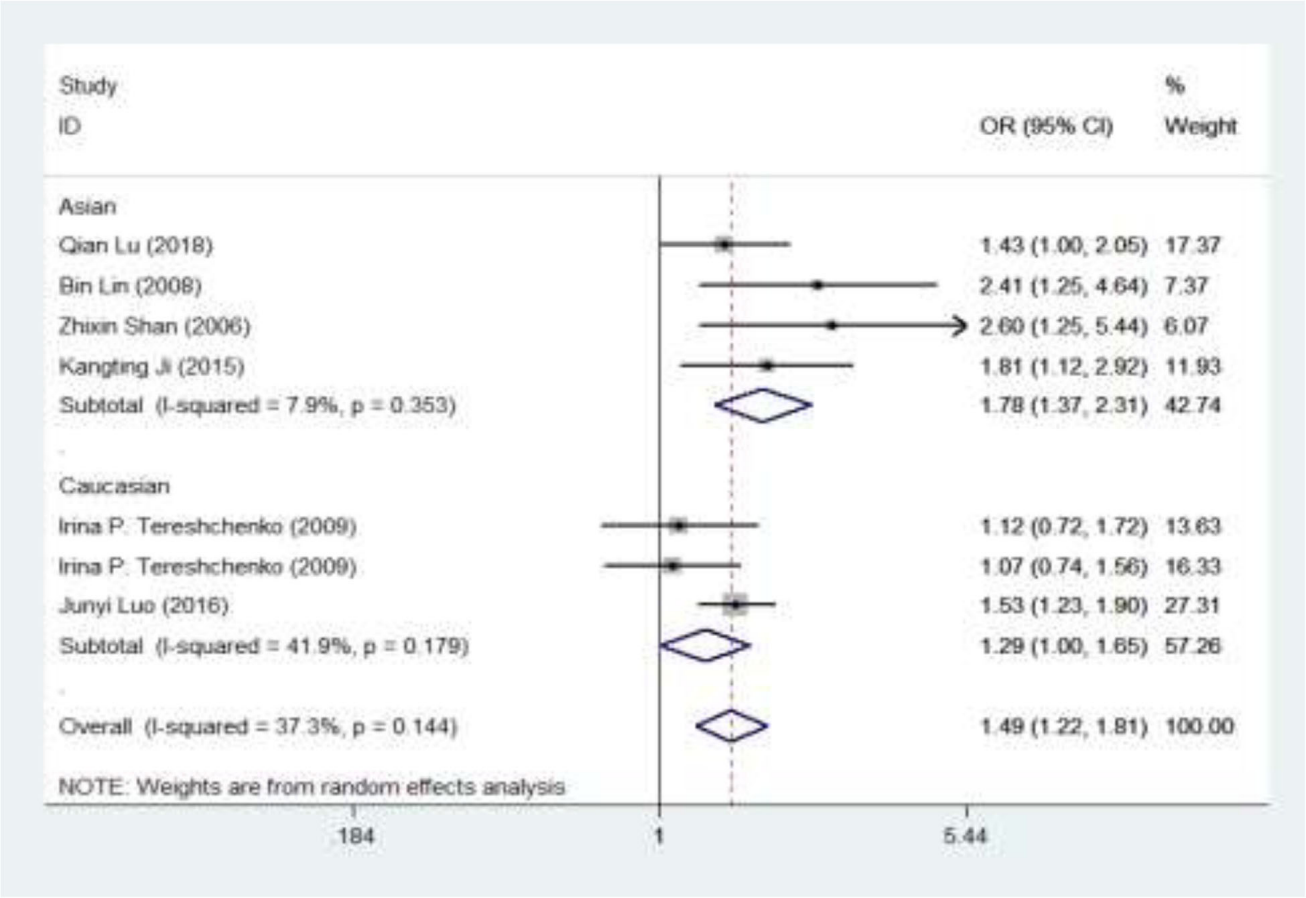
Forest plot of MIF-173C/G rs755622 in subgroup analysis for allele comparison (C vs. G)

Data collected from the six studies have been summarized for gene analysis (Table 1). It was found that the gene polymorphism in healthy controls and patients with CAD was in HWE, which indicated that the gene frequency and genotypic frequency were balanced. We not only studied *MIF* -173C/G homozygotes and heterozygotes with CAD, but also incorporated dominant, recessive, and additional models to observe the relationships between these models and the risk of CAD, so as to conduct a more comprehensive meta-analysis. Thus, we compared allelic, homozygote, heterozygote, dominant, recessive, and additive models. Based on analysis of the total population, the risk for CAD was found to be increased by 48.9% under the allelic model (C/G: odds ratio [OR] = 1.489, 95% confidence interval [CI] = 1.223–1.813, *P* < 0.001), by 123.2% under the homozygote model (CC/GG: OR = 2.232, 95% CI = 1.559–3.197, *P* < 0.001), and by 106.5% under the recessive model (CC/CG + GG: OR = 2.065, 95% CI = 1.454–2.933, *P* < 0.001). The risk for CAD was reduced by 43.5% under the heterozygote model (CG/CC: OR = 0.565, 95% CI = 0.39–0.82, *P* = 0.003) and by 31.8% under the dominant model (GG/CG + CC: OR = 0.682, 95% CI = 0.535–0.871, *P* = 0.002). The additive model (CG vs. CC + GG: OR = 1.242, 95% CI = 0.876–1.762, *P* = 0.223) had no statistical significance (Fig. 2, Supplementary Figure S2-S6, Table 2).

**Table 2:**
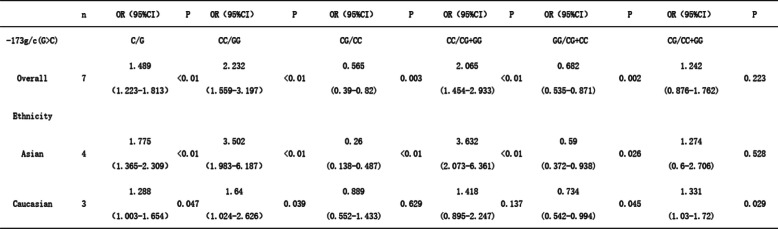
Summary of polled odds rations in the meta-analyis

To clarify the source of heterogeneity and reduce the heterogeneity of the conclusions, we next conducted subgroup analysis in subgroups according to ethnicity.

### Association between *MIF* -173 C/G and CAD susceptibility in the Asian population

In the Asian population, the risk for CAD was increased by 77.5% under the allelic model (C/G: OR = 1.775, 95% CI = 1.365–2.309, *P* < 0.001), by 250.2% under the homozygote model (CC/GG: OR = 3.502, 95% CI = 1.983–6.187, *P* < 0.001), and by 263.2% under the recessive model (CC/CG + GG: OR = 3.632, 95% CI = 2.073–6.361, *P* < 0.001), whereas the risk was reduced by 74.0% under the heterozygote model (CG/CC: OR = 0.260, 95% CI = 0.138–0.487, *P* < 0.001) and by 41.0% under the dominant model (GG/CG + CC: OR = 0.590, 95% CI = 0.372–0.938, *P* = 0.026) (Table 2).

### Association between *MIF* -173 C/G and CAD susceptibility in the Caucasian population

In the Caucasian population, the risk for CAD was increased by 28.8% under the allelic model (C/G: OR = 1.288, 95% CI = 1.003–1.654, *P* = 0.047), by 64.0% under the homozygote model (CC/GG: OR = 1.640, 95% CI = 1.024–2.626, *P* = 0.039), and by 33.1% under the additive model (CG/CC + GG: OR = 1.331, 95% CI =1.03–1.72, *P* = 0.029), and the risk was reduced by 26.6% under the dominant model (GG/CG + CC: OR = 0.734, 95% CI = 0.542–0.994, *P* = 0.045). There was no significant effect under the recessive (CC/CG + GG: OR = 1.418, 95% CI = 0.895–2.247, *P* = 0.137) and heterozygote (CG/CC: OR = 0.889, 95% CI = 0.552–1.433, *P* = 0.629) models (Table 2).

### Sensitivity and publication bias analyses

**Fig. 3:**
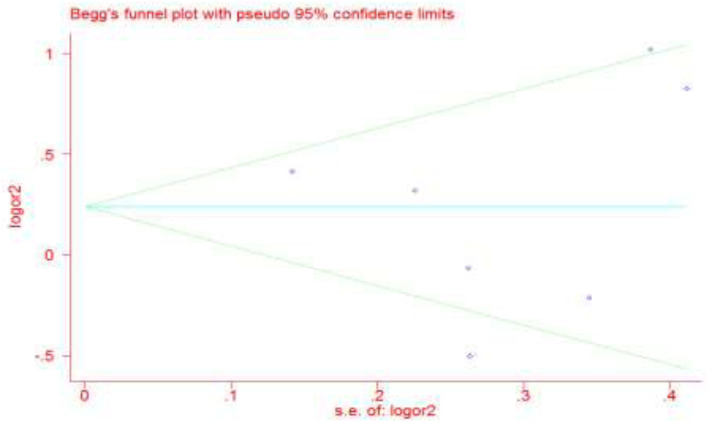
Begg’s funnel plot for publication bias analysis for MIF-173C/G polymorphism rs755622 in homozygotes comparison (CC

To investigate the stability of the meta-analysis, we used a sensitivity analysis. The OR value was not affected by a sequential exclusion of any of the six studies using the six genetic models. Although funnel plots are often used to evaluate publication bias, it has some unavoidable limitations, such as the need for different sample sizes for various studies and the fact that the presence of artificial subjective judgments affect the final decision. Therefore, we used Egger’s linear regression test to assess publication bias for *MIF* -173C/G and CAD risk. Egger’s test indicated the presence of sample bias (C vs. G: t = − 0.15, *P* = 0.89), which we considered to be due to the fact that the sample size was not sufficiently large; however, this did not affect the results of the analysis (Fig. 3).

## Discussion

CAD, a chronic arterial wall disease, is not simply due to an accumulation of lipids in the body, but is an inflammatory disease in response to an injury, in which inflammatory cells and mediators are involved in plaque formation. *MIF* was the first cytokine to be considered an important mediator of chronic inflammatory and autoimmune diseases, mediating the generation of inflammatory cells. *MIF* is an important proinflammatory factor and chemokine. It can recruit inflammatory immune cells, such as macrophages and T lymphocytes, to participate in the inflammatory response at atherosclerotic plaques [26, 27]. It can also stimulate macrophages and lymphocytes to secrete inflammatory factors, including IL-6, IL-8, TNF-α, and intracellular adhesion factor, which significantly enhance the inflammatory response at atherosclerotic plaques [10]. *MIF* can be expressed at low levels in vascular smooth muscle cells and vascular endothelial cells, but when atherosclerotic plaques occur in the blood vessels, the production of *MIF* increases rapidly, suggesting it may be involved in the occurrence of atherosclerosis. In recent years, *MIF* has been explored in genetic and molecular functional studies [28]. The *MIF* -173C allele establishes a hypothetical binding site for transcription factor activating enhancer binding protein 4, which may increase *MIF* gene and protein expression [14].

The MONICA/KORA Augsburg study reported that the *MIF* rs755622 C allele increased the susceptibility to juvenile idiopathic arthritis, systemic lupus erythematosus, and celiac disease associated with severe ulcerative colitis [17, 20, 26, 29]. The hypothesis that *MIF* rs755622 polymorphisms regulate *MIF* levels only in certain conditions, e.g., in response to infection, trauma, or autoimmunity, was confirmed in a cardiopulmonary bypass study [30]. Only *MIF* -173C allele carriers receiving revascularization and cardiopulmonary bypass had elevated systemic *MIF* levels, indicating genotype changes in *MIF* expression affected by trauma and injury. Alternatively, rs755622 may be mainly related to localized *MIF* expression, that is, *MIF* expression can be increased in certain cell types or tissues, such as atherosclerotic lesions. However, there is currently no evidence supporting this speculation.

The mechanistic link between *MIF* -173C alleles and disease remains unclear, as most studies focused on genotyping data and did not evaluate circulating *MIF* levels. Nevertheless, we can unravel the correlation between *MIF* rs755622 polymorphism and CAD based on published articles. One study showed that *MIF* -173C was associated with an increased risk CAD in German women, based on an average follow-up period of 10 years [31]. In the Han Chinese too, *MIF* -173C has been identified as a CAD risk factor [17, 19]. Yang et al. reported that plasma *MIF* levels may be used to predict the severity of coronary lesion [32]. Meanwhile, *MIF* -173G/C polymorphism influences the plasma levels of *MIF* and TNF-α in some inflammatory disorders [33, 34]. However, the precise regulatory mechanisms of this association remain unclear. Relevant studies suggest that the *MIF* rs755622 G/C polymorphism may play a critical role in the etiology of coronary artery lesions and may have predictive value for their severity. The analysis of more *MIF* polymorphisms may help to identify individuals with potential CAD risk, and identifying targeted *MIF* variants in CAD patients may be beneficial for risk stratification and management.

In our systematic meta-analysis, including six studies comprising approximately 2700 participants, *MIF* -173C/G was associated with CAD risk in the overall population and in two ethnic subgroups (Asians and Caucasians). CAD risk was increased in the allelic, recessive, and homozygote models, whereas it was decreased in the dominant and heterozygote models. To identify and reduce the source of heterogeneity, we analyzed the Asian and Caucasian populations separately. We could conclude that people with *MIF* -173C were more likely to develop CAD, which might be related to increased *MIF* expression and production, which aggravated the inflammatory reaction, leading to the occurrence and development of CAD. There are several potential causes of the observed heterogeneity. First, study characteristics, including the source population for the case-control groups, race, research design, CAD subtypes, sample size, genotyping methods and specific typing can differ among studies and may explain the differences in subgroup analysis. Second, ethnic groups are genetically different, and clinical manifestations depend on factors such as age, sex, obesity, hypertension, and diabetes, which may be related to CAD heterogeneity [35]. Third, different populations have different lifestyles, including eating habits, exercise patterns, which may affect CAD onset and progression.

Our meta-analysis had some strengths. First, a comprehensive meta-analysis to provide substantial evidence of the association between *MIF* -173C/G and CAD, which would allow a more accurate judgment of this gene polymorphism in the treatment of human CAD, had not been conducted to date. Second, we selected Chinese and foreign studies, including two different ethnicities, for which we conducted subgroup analyses, which allowed us to find the source of heterogeneity and further assess the risk of *MIF* -173C/G for CAD in these two populations. In contrast, our study had several limitations. First, it included only six studies. Therefore, additional information from large-cohort studies on *MIF* -173C/G and CAD is required to reduce the publication bias. Second, numerous factors can influence the occurrence and development of coronary heart disease, and we did not consider other polymorphisms in *MIF*, such as -794CATT_5–8_, or in other related genes. Third, despite the inclusion of different ethnic groups, populations may have different habits, including different eating and lifestyle habits, which should be considered more carefully in future studies. Fourth, more research is needed to confirm the relevance of -173 C/G to the risk of CAD and to clarify the underlying mechanism.

## Conclusion

Our meta-analysis demonstrated that *MIF* -173C/G polymorphism was associated with CAD susceptibility. *MIF* -173C/G may regulate and increase the risk of CAD by increasing *MIF* secretion. Our findings may help to understand the effects of CAD-related genes in total the population and in different ethnic groups, and lay a foundation for the prevention of coronary heart disease in the future.

## Supplementary information

**Additional file 1 Supplementary Table S1**. Scale for Methodological Quality Assessment.

**Additional file 2 Supplementary Figure S2** Forest plot of MIF -173C/G rs755622 in subgroup analysis for homozygote model comparison (CC vs.GG). **Supplementary Figure S3** Forest plot of MIF -173C/G rs755622 in subgroup analysis for recessive model comparison (CC vs.CG + GG). **Supplementary Figure S4** Forest plot of MIF -173C/G rs755622 in subgroup analysis for heterozygote model comparison (CG vs.CC). **Supplementary Figure S5** Forest plot of MIF -173C/G rs755622 in subgroup analysis for dominant model comparison (GG vs.CG + CC). **Supplementary Figure S6** Forest plot of MIF -173C/G rs755622 in subgroup analysis for additive model comparison (CG vs.CC + GG).

## Data Availability

All data generated or analyzed during this study have been included in this published article.

## Abbreviations

MIF: Macrophage migration inhibitory factor
CAD: Coronary artery disease
IL: Interleukin
TNF: Tumor necrosis factor
HWE: Hardy–Weinberg equilibrium
OR: Odds ratio
CI: Confidence interval
