# Supplementary material for: MIF -173G/C (rs755622) polymorphism modulates coronary artery disease risk: evidence from a systematic meta-analysis
Source: BMC Cardiovasc Disord. 2020 Jun 19;20:300. doi: 10.1186/s12872-020-01564-4 (PMC7304150; doi:10.1186/s12872-020-01564-4)
Supplement: Supplementary file 1 — Additional file 1 Supplementary Table S1. Scale for Methodological Quality Assessment. [file 12872_2020_1564_MOESM1_ESM.pdf]

Supplementary Table S1. Scale for Methodological Quality Assessment

Criteria	Score
1. Representativeness of cases	
CAD diagnosed according to acknowledged criteria	2
Mentioned the diagnosed criteria but not specifically described	1
Not mentioned	0
2. Source of controls	
Population or community based	3
Hospital-based CAD-free controls	2
Healthy volunteers without total description	1
CAD-free controls with related diseases	0.5
Not described	0
3. Sample size	
>300	2
200–300	1
<200	0
4. Quality control of genotyping methods	
Repetition of partial/total tested samples with a different method	2
Repetition of partial/total tested samples with the same method	1
Not described	0
5. HWE	
HWE in control subjects	2
Hardy–Weinberg disequilibrium in control subjects	1

CAD coronary artery disease, HWE Hardy–Weinberg equilibrium.
