# Supplementary material for: MIF -173G/C (rs755622) polymorphism modulates coronary artery disease risk: evidence from a systematic meta-analysis
Source: BMC Cardiovasc Disord. 2020 Jun 19;20:300. doi: 10.1186/s12872-020-01564-4 (PMC7304150; doi:10.1186/s12872-020-01564-4)
Supplement: Supplementary file 2 — Additional file 2 Supplementary Figure S2 Forest plot of MIF -173C/G rs755622 in subgroup analysis for homozygote model comparison (CC vs.GG). Supplementary Figure S3 Forest plot of MIF -173C/G rs755622 in subgroup analysis for recessive model comparison (CC vs.CG + GG). Supplementary Figure S4 Forest plot of MIF -173C/G rs755622 in subgroup analysis for heterozygote model comparison (CG vs.CC). Supplementary Figure S5 Forest plot of MIF -173C/G rs755622 in subgroup analysis for dominant model comparison (GG vs.CG + CC). Supplementary Figure S6 Forest plot of MIF -173C/G rs755622 in subgroup analysis for additive model comparison (CG vs.CC + GG). [file 12872_2020_1564_MOESM2_ESM.docx]

####
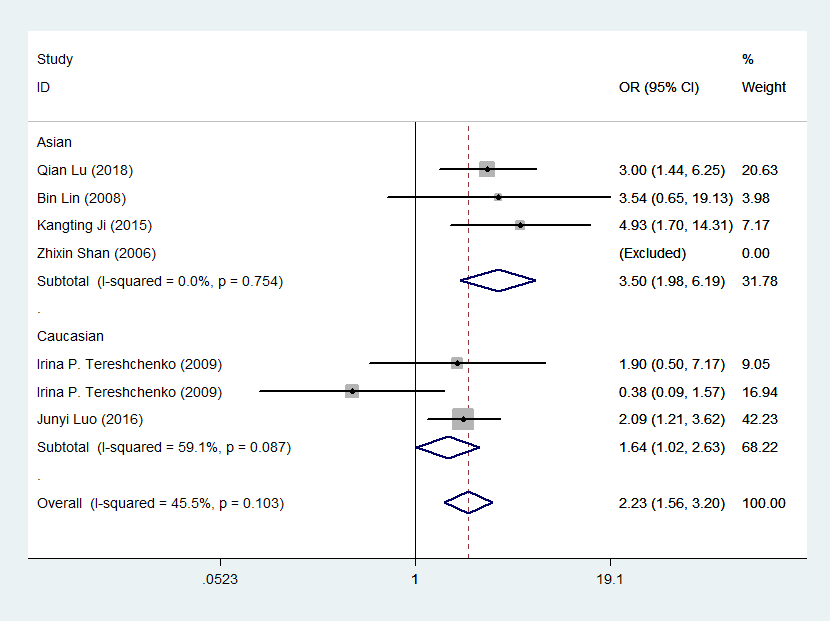


**Supplementary Figure S2**

Forest plot of MIF -173C/G rs755622 in subgroup analysis for homozygote model comparison (CC vs. GG).

####
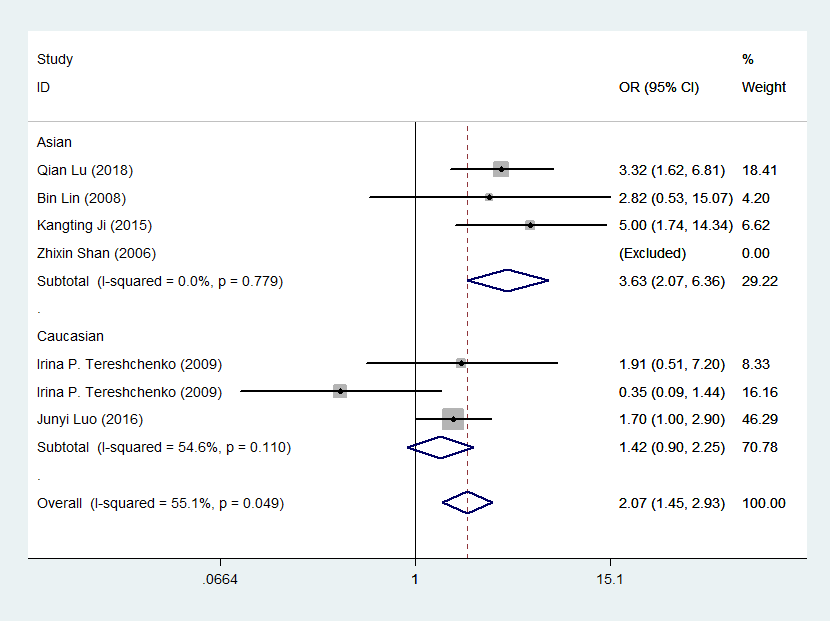


**Supplementary Figure S3**

Forest plot of MIF -173C/G rs755622 in subgroup analysis for recessive model comparison (CC vs. CG+GG).

####
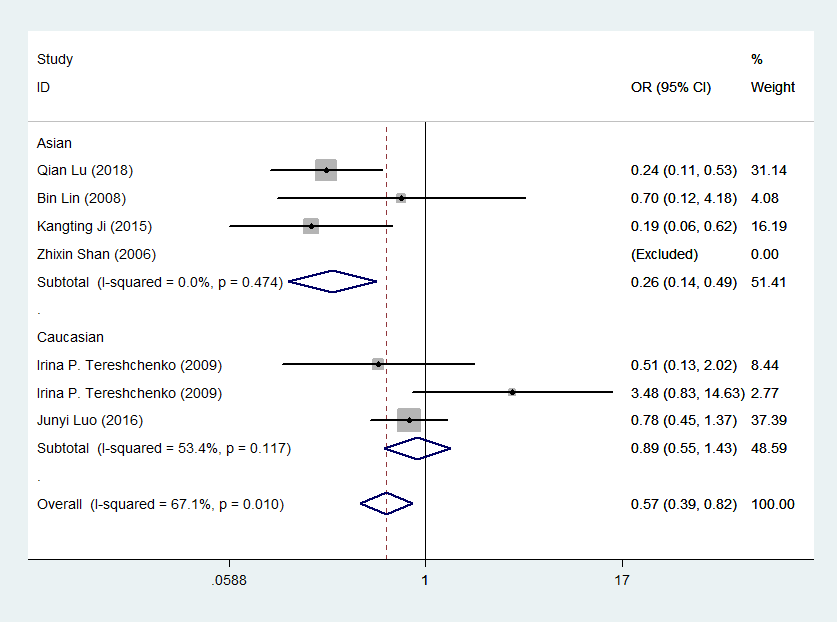


**Supplementary Figure S4**

Forest plot of MIF -173C/G rs755622 in subgroup analysis for heterozygote model comparison (CG vs. CC).

####
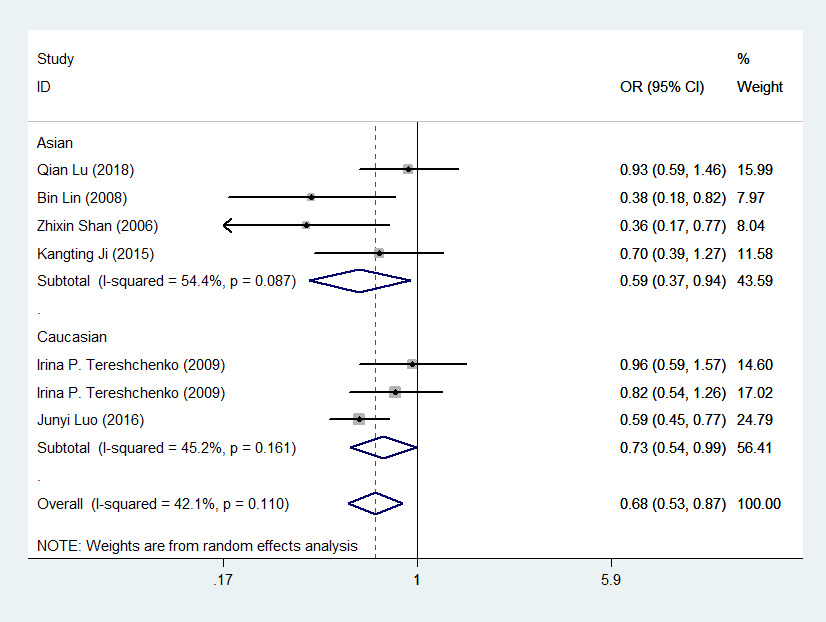


**Supplementary Figure S5**

Forest plot of MIF -173C/G rs755622 in subgroup analysis for dominant model comparison (GG vs. CG+CC).


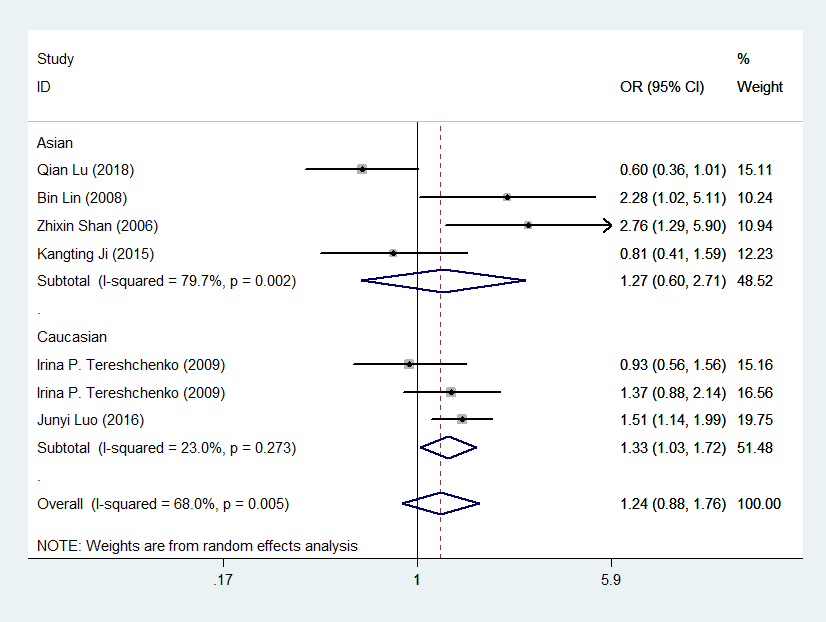


**Supplementary Figure S6**

Forest plot of MIF -173C/G rs755622 in subgroup analysis for additive model comparison (CG vs. CC+GG).
